# Editorial: Blindness, Light, and the COVID-19 Pandemic

**DOI:** 10.3389/fvets.2021.689981

**Published:** 2021-06-10

**Authors:** Andres M. Perez

**Affiliations:** College of Veterinary Medicine, University of Minnesota, Minneapolis, MN, United States

**Keywords:** COVID-19, SARS-CoV-2, control, pandemia, veterinary

In 1995, Nobel Laureate Jose Saramago published *Blindness* (*Ensaio sobre a cegueira* in Portuguese), a novel that describes the effects of a mass epidemic of blindness. Unlike Saramago's book, in which the cause of such sudden condition remained unexplained, the agent causing the most devastating human pandemic in recent history, referred to as the severe acute respiratory syndrome coronavirus 2 (SARS-CoV-2), has been fully characterized shortly after its first identification in late 2019 in Wuhan, China. Coronaviruses are relatively novel threats to public health. However, veterinarians have a long-standing experience in fighting diseases caused by this family of RNA viruses. Furthermore, mass epidemics have recently affected animals more frequently than humans and for that reason, many veterinarians have had the chance to experience the complexities associated with dealing with emergencies that resemble the challenges associated with the coronavirus disease (COVID-19) pandemic.

In response to this health crisis, probably for the first time in history, the scientific community was in the spotlight. However, public attitudes and opinions toward science were heavily polarized and influenced by political, social, and philosophical views. While many looked at science as a source of answers and a resource for data and information that enable better understanding, and ultimately control and prevention of the disease, there was also miscommunication, skepticism, and even social revelry. In the middle of such complex social, economical, and political scenarios, and while the scientific community engaged in an unprecedented race for discovery, from the onset of the pandemic, veterinarians placed themselves at the forefront of that fight. Veterinary Clinics and hospitals were adapted to fulfill protocols and mitigate risks while still taking care of their mission, veterinary laboratories were adapted to aid with the diagnosis and molecular characterization of the SARS-CoV-2, human and financial resources were shifted to support research aimed at helping with the control and prevention of the disease. To honor the efforts of veterinarians around the globe, the World Veterinary Association (WVA) dedicated the 2021 World Veterinary Day (April 24) to celebrate the work of veterinarians to protect animal and human health during the COVID-19 pandemic (https://www.worldvet.org/news.php?item=465). This Research Topic by Frontiers in Veterinary Science joins that celebration, bringing together a collection of 12 scientific papers representing stories, opinions, perspectives, and research results that illustrate the impact that the COVID-19 pandemic has caused on veterinary sciences and veterinarians. Papers have been grouped into three major areas, illustrating how the pandemic has (a) impacted our perspectives for veterinary public health and one health, (b) affected animal welfare and the operation of veterinary services, and (c) promoted novel research initiatives and opportunities, respectively.

## (A) Veterinary Public Health and One Health

Although the benefits of the coexistence of animals and humans have traditionally exceeded the negative consequences of such interaction, in recent decades, human population growth and efforts to alleviate poverty and hunger have led to an increase in the magnitude and complexity of such interactions, affecting widespread and disparate geographies and a variety of animal species. Consequently, there was a dramatic increase in the risk for the emergence and transmission of diseases in the human-animal interface. There is an urgent need to search for, identify, and characterize potential sources of emerging pathogens at the animal-human interface and to develop strategies to prevent or mitigate the risk for future pandemics (Magouras et al.). The extent and complexity of the human-animal interaction have also been affected, in some places, by an increase in the legal and illegal trade of wildlife for consumption, which, in addition to increasing the risk for disease transmission, has impaired access to resources for native communities around the world, which typically rely on wild meat to meet their nutritional requirements (Walzer). However, silver bullets do not exist and solutions are difficult to design and implement; indeed, there is a risk that restrictive measures to trade, imposed in an attempt to reduce the likelihood of diseases spreading, would affect food security further, adding to the damage that the pandemic has caused to global food access (Mardones et al.).

## (B) Animal Welfare and Operation of Veterinary Services

COVID-19 outbreaks have had devastating consequences for some activities, particularly those conducted in confined spaces in which workers are located close to each other, such as meat processing plants. The capacity of pig processing plants in the U.S. was reported to have decreased by 45% at a given time during the epidemic, representing a daily reduction of ~250,000 animals in the country's capacity to slaughter pigs. The situation severely affected animal welfare, in the form of longer transportation times to process pigs in plants that were still active, culling of animals in farms, and the potential environmental impact associated with the disposal of those carcasses (Marchant-Forde and Boyle). In response to the crisis, in many countries, multisectoral groups were established in an attempt to mitigate the impact of the pandemic on animal welfare. For example, a group of organizations in Australia outlined recommendations aimed at protecting animal welfare in the country and promoting similar actions in other regions (Baptista et al.).

Veterinary clinics, hospitals, and laboratories have also been affected by the pandemic, with regular function and activity disrupted in many complex ways. Challenges included, for example, partial or complete shutdowns, interrupted courier services, disruptions in workflow and diagnostic testing, and the need to adapt laboratories to new physical distancing practices, protocol development or enhancement for handling samples from high risk or susceptible species, and fulfilling requirements for pre-test permission approval from state and federal veterinary agencies (Stokol et al.).

## (C) Emerging Opportunities and Consequences for Veterinary Research

On the other hand, the COVID-19 pandemic also inspired and promoted research initiatives emerging from veterinary sciences throughout the world, with the objective of aiding control of the emergency under a One Health umbrella. In the interface of animal and public health, there is an opportunity for the knowledge and experiences emerging from veterinary sciences to accelerate response and preparedness against COVID-19 and other potential emerging threats (Mobasheri).

One potential area of collaboration is rooted in the basic and applied research conducted for decades in the veterinary field to develop antivirals and immune modulators to help control the diseases caused by coronaviruses in animals. For example, recombinant bovine gamma interferon (rbIFN-λ) was found to be effective in preventing SARS-CoV-2 infection in VERO cells (Cardoso et al.). Similarly, animal models of coronavirus infections can help understand the pathogenesis and implications of the disease in humans. For example, neurological signs have been reported in human cases of COVID-19, while, recently, there have been promising results in the use of antiviral drugs for the treatment of the neurological form of coronavirus infection in cats (Dickinson). In addition to animal models, epidemiological models originally developed for animal diseases were adapted to help explain and predict the spread of COVID-19 in human populations (Halasa et al.).

Successful initiatives to support this response through a collaborative One Health approach were launched, including, for example, the parameterization of mathematical models of COVID-19 spread in Ireland, leverage of public and veterinary epidemiology resources to support the response to the pandemic in Australia, and multinational collaboration to create a platform for knowledge exchange in sub-Saharan Africa (Häsler et al.). In a broad context, many lessons learned from the management of animal health emergencies could have helped and should be adopted in the future, anticipating further global failure, which has already been experienced in addressing the COVID emergency, stressing an urgent to revisit a global strategy for implementation of the One Health agenda (Enticott and Maye).

In conclusion, similar to the character that escaped blindness, who was able to see during the epidemic described in Saramago's fictional novel, we expect that the collection here illustrates the light that veterinary sciences may bring in the form of reflection, and the generation of the foundational knowledge required to develop tools and strategies for fighting one of the most impactful health challenges experienced in our recent history, while increasing preparedness and mitigating the risk for, and impact of, future global emergencies.

## Author Contributions

AP was the sole author of this manuscript.

## Conflict of Interest

The author declares that the research was conducted in the absence of any commercial or financial relationships that could be construed as a potential conflict of interest.

